# Parent–Child Mismatch in Educational Expectations and Depressive Symptoms among Chinese Adolescents

**DOI:** 10.3390/healthcare12171792

**Published:** 2024-09-07

**Authors:** Yueyun Zhang, Meng Jiang

**Affiliations:** School of Social Sciences, Harbin Institute of Technology, Harbin 150001, China

**Keywords:** depressive symptoms, parent–child mismatch in educational expectations, Chinese adolescents

## Abstract

Background: The roles of both parents’ and children’s educational expectations in shaping adolescent depressive symptoms have increasingly been discussed, yet in a separate manner. To date, few studies have associated parent–child mismatch in educational expectations with depressive symptoms, and less is known about the variation in the association across gender (male vs. female), educational level (primary vs. secondary), and region status (urban vs. rural) in the Chinese educational setting. Methods: Respondents were from a nationally representative sample of adolescent students in China (sample size: 1844; age range: 10–15 years). Parent–child mismatch in educational expectations included three categories: (1) “match”, (2) “mismatch—parent higher”, and (3) “mismatch—parent lower”. Regression analysis with inverse propensity-score weighting was employed to estimate the effect of parent–child mismatch as to educational expectations on depressive symptoms, and stratified analysis was used to examine the variation of the effect by gender, educational level, and region. Results: Compared with the “match” group, the “mismatch—parent higher” group had significantly higher levels of depressive symptoms. Furthermore, the pattern remained consistent between boys and girls, but differed significantly by adolescents’ educational level and region status. Specifically, the pattern was more pronounced in the primary school and urban subsamples. Conclusions: Findings in this study indicated that educators and policymakers can develop tailored strategies to alleviate depressive symptoms among the “mismatch—parent higher” group, and especially for those children from primary schools and urban areas.

## 1. Introduction

Depressive symptoms are one of the most common mental health problems [1], with an upward trend evident from childhood to adolescence [2,3] and they often peak in mid-adolescence, at around the age of 15 [4,5]. China is no exception. A recent study based on a nationally representative sample of Chinese adolescents has found that the prevalence of depression was about 23.2 percent [6]. Another recent estimate has illustrated that Chinese adolescents’ depression has increased by at least 0.76 standard deviations from 1989 to 2018 [7]. Social media use and other psychological factors, such as childhood trauma, are contributing to depressive symptoms in adolescents [8,9]. Depressive symptoms during adolescence not only influence children’s subsequent lives and academic performance, but also have long-term impacts on life, work, and physical well-being in later life [10]. Therefore, gaining a profound understanding of the factors influencing adolescent depressive symptoms becomes a paramount concern in effectively alleviating this condition.

Empirical studies have recently shown that educational expectations, which have long been discussed in the educational literature, could significantly affect students’ mental health. Notably, previous studies have separately examined the impacts of parents’ or children’s educational expectations on depressive symptoms. For example, one study based on a sample of adolescents in grades 7–12 showed that as parental educational expectation increases, the prevalence of depression among adolescents decreases [11]. Another study, focused on adolescents in Sweden, demonstrated that higher academic expectations among adolescents were associated with having fewer mental health problems [12].

Although previous research has paid much attention to both parents’ and adolescents’ educational expectations [13,14], a large proportion of adolescents may experience a mismatch between parental expectations and their own [15,16]. Such a parent–child mismatch in educational expectations may lead to negative psychological consequences. The identity control theory seems to be useful for understanding the association between parent–child mismatch in educational expectations and adolescent mental health. Identity control theory explains how individuals maintain and adjust their self-identity through social feedback. The theory states that individuals tend to seek social feedback from others that is consistent with the way they see themselves; this is known as “identity verification”. According to this theoretical perspective, inconsistencies between external reflective evaluations and internal identity standards can elicit negative emotional responses in individuals, such as depression and anxiety [17]. When there is a parent–child mismatch in educational expectations, the child may experience a failure of authentication, which can lead to negative emotions. In addition, individuals receive information through a feedback loop and accordingly adjust their behavior and cognition to align with social expectations and personal goals. When there is a parent–child mismatch in educational expectations, this mismatch can break the normal feedback loop, causing the child to experience barriers in academic and personal development and increasing the child’s depressive symptoms [18]. Social identity theory can also help us understand this relationship. The theory suggests that an individual’s self-concept is partly derived from the group to which they belong, and that this sense of belonging can significantly influence an individual’s psychology and behavior [19]. When adolescents’ self-identity is at odds with their parents’ expectations of them, conflict and stress can arise, and this stress can sometimes lead to the appearance of depressive symptoms [20,21].

Several previous studies have examined the impacts of parent–child mismatch in educational expectations on adolescent students, but these impacts have been subject to a narrow focus and limited to academic performance and cognitive ability, with little attention given to mental health outcomes such as depression. For example, one previous study found that children’s cognitive ability tended to be lower when the parental educational expectation was higher than the child’s own educational expectation [22]. Another study examined the relationship between parent–child mismatch in educational expectation and academic achievement, showing that higher levels of parent-child agreement on educational expectations promote student achievement, and higher levels of parent-child disagreement on expectations inhibit student achievement [23]. To our knowledge, few studies have directly linked a parent–child mismatch in educational expectation to adolescent depressive symptoms, the latter of which, as mentioned above, are currently quite common in China.

Notably, a growing body of literature has noticed that such a mismatch may be associated with increased levels of mental health problems, but the empirical evidence remains limited. For example, studies have examined the phenomenon in American and Swedish populations [15,23,24]. But not enough attention has been paid to China. Although there are some studies using China as a background, their samples have been relatively simple and unrepresentative [16,25]. Moreover, studies on heterogeneity are also insufficient [26].

This study aims to address this gap using data from a nationally representative sample of Chinese adolescents. Extending upon the prior literature, we obtained parent–child mismatch in educational expectations data by cross-classifying parents’ and children’s educational expectations, yielding three categories: (1) the “match”, (2) the “mismatch—parent higher”, and (3) the “mismatch—parent lower”. Regression analysis with inverse propensity-score weighting was employed to estimate the effect of parent–child mismatch in educational expectations on depressive symptoms, and stratified analysis was used to examine the variation of the effect by gender, educational level, and region (rural vs. urban). Taken together, this study would contribute to the previous literature by highlighting the specific role of parent–child mismatch in educational expectations in affecting adolescent depressive symptoms, using empirical evidence from China. Findings in this study may have important implications for societies that are witnessing a common occurrence of parent–child mismatch in educational expectations and are concerned about its impact on the mental health of the adolescent students.

## 2. Methods

### 2.1. Participants

**Figure 1:**
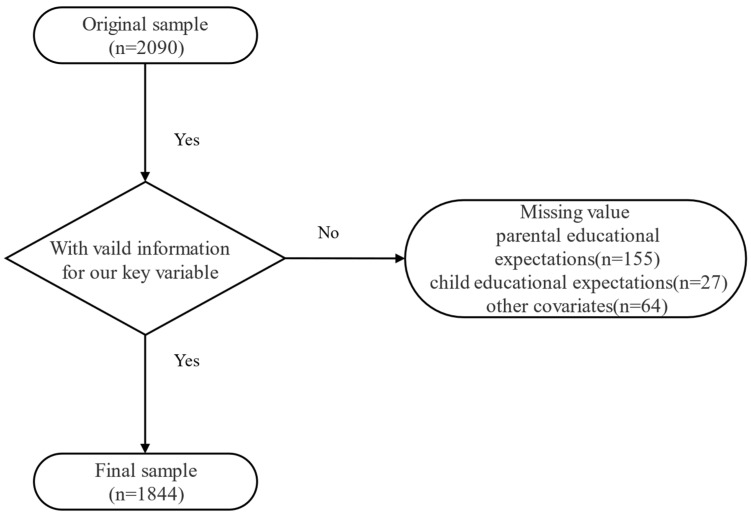
Flow chart of sample selection with inclusion and exclusion criteria.

The participants were taken from the China Family Panel Studies (CFPS), a large-scale and nationally representative survey project led by the China Social Science Survey Center at Peking University. The baseline survey was conducted in 2010, and follow-up surveys were then conducted every two years. This study used data from the 2020 cross-sectional survey and focused specifically on the sample of adolescents aged 10–15 years. The original sample size was 2090. We excluded respondents with missing values for parental educational expectations (n = 155), child expectations (n = 27), and other covariates (n = 64). Therefore, our final analytic sample consisted of 1844 adolescents. Figure 1 illustrates the steps we followed to obtain the final sample.

### 2.2. Measure

#### 2.2.1. Depressive Symptoms

Depressive symptoms were evaluated using an 8-item scale adapted from the Center for Epidemiological Studies Depression Scale (CES-D) [27]. Respondents were asked to rate their symptoms based on eight items, using a 4-point Likert scale ranging from 0 (almost never/less than 1 day) to 4 (most of the time/5–7 days). Two items asking about positive symptoms were reverse-coded. Then, the eight items were summed up, obtaining a total score ranging from 0 to 24. As such, higher CES_D8 scores would indicate greater levels of depressive symptoms. The 8-item CES-D has been seen as a reliable and valid abbreviated scale for Chinese adolescents [28,29], and has increasingly been used in depression studies [30,31]. In the current study, the internal consistency of the 8-item CES-D was high, with a Cronbach’s alpha of about 0.736.

#### 2.2.2. Parent–Child Mismatch

Parent–child mismatch in educational expectations is the key explanatory variable. It was assessed by comparing parents’ and children’s educational expectations. Parental educational expectations were measured using the following question: “What is the minimum level of education you hope your child will complete?” Children’s educational expectations were measured using the CFPS Individual Self-Response Questionnaire, which includes the following question: “What is the minimum level of education you think you should complete?” Both sets of responses included options ranging from “primary school” to “doctorate” and were collapsed into four broad categories: below senior high school, senior high school, college, and postgraduate. We then constructed values for parent–child mismatch in educational expectations by cross-classifying parents’ and children’s educational expectations, yielding three categories: (1) the “match”, (2) the “mismatch—parent higher”, and (3) the “mismatch—parent lower”. Where necessary, we made elaborations within the “mismatch—parent higher” group according to parental educational expectations of “senior high school”, “college”, and “postgraduate”. Table 1 shows the classification process.

#### 2.2.3. Control Variables

We adjust for sociodemographic and family contexts that are correlated with both parent–child mismatch and depressive symptoms [32,33], and which may therefore confound the relationship between parent–child mismatch and depressive symptoms. Control variables included respondents’ personal characteristics and family background. Personal characteristics included gender (male = 0; female = 1), age, grade, and academic achievement. Academic achievement was represented by the student’s average scores in Chinese and mathematics, respectively. Family background included parental education, family economic condition, and region (rural = 0; urban = 1). Parental education was measured by taking the highest level of education reported for either the father or the mother. Family economic condition was measured by the logarithm of family income per capita.

### 2.3. Statistical Analysis

All analyses were conducted using Stata for Windows, version 12.0 (StataCorp, College Station, TX, USA). Descriptive statistics were used to report the means and standard deviations for continuous variables, such as depressive symptoms, and the frequencies and percentages for categorical variables, such as parent–child mismatch in educational expectations. Regression analyses were conducted to assess the effect of parent–child mismatch in educational expectations on adolescent depressive symptoms. Given that the previous literature indicates that parent–child mismatch in educational expectations is influenced by individual characteristics and family background, we employed the inverse propensity-score weighting approach to mitigate the confounding effects of these factors. The propensity score represents the probability of each case being in a given sub-sample, based on the covariates. This method offers three primary advantages. First, it equalizes the distribution of different groups across a range of covariates through weighting, thereby making the groups truly comparable. Second, it minimizes sample deletion, thereby maximizing the information contained in the full sample. Third, it reduces the large number of covariates in a multiple regression model to a single propensity score, resulting in a more concise presentation of the results [34].

After reporting results based on the full sample, we conducted a stratified analysis to investigate whether and how the effect of parent–child mismatch in educational expectations on depressive symptoms varies by adolescents’ gender, educational level, and region.

## 3. Results

### 3.1. Descriptive Results

Table 2 shows descriptive statistics for the overall sample and, separately, for the “match”, “mismatch—parent lower”, and “mismatch—parent higher” subsamples. Overall, the depressive symptoms score was 4.27. In parent–child mismatch in educational expectations, 55.86% were matched, 13.39% were “mismatch—parent lower”, and 30.75% were “mismatch—parent higher”. In terms of “mismatch—parent higher”, 7.76% of the parental educational expectations were at the senior high school level, 64.20% were at college level, and 28.04% were at the postgraduate level. Depressive symptoms showed a significant difference among the three groups of parent–child mismatches in educational expectations (*p* < 0.001), and the “mismatch—parent higher” group had the most severe psychological punishment.

### 3.2. Regression Results

Table 3 presents the results of regression models predicting depressive symptoms by parent–child mismatch. Each model is weighted with propensity score weights to attenuate potential bias due to the confounding effects of respondents’ personal characteristics such as gender, age, grade, and academic achievement, and family characteristics such as parental education, family economic status, and region. Model 1 in Table 3 shows that adolescents in the “mismatch—parent higher” group had the highest level of depressive symptoms, which was 0.59 points higher than in the “match” group. To further refine the results, the study explored the effects of different levels of parental educational expectations in the “mismatch—parent higher” group on depressive symptoms. Focusing on comparisons within the group “mismatch—parent higher”, Model 2 in Table 3 shows that the highest score for depressive symptom is in the senior high school group, followed by the college group, which are, significantly as to both, 2.25 and 0.65 points higher than in the “match” group, respectively.

### 3.3. Stratified Regression Results

Table 4, Table 5 and Table 6 show the regression results for parent–child mismatch in educational expectations and depressive symptoms among adolescents of different genders, educational levels, and regions, respectively. All regressions were obtained after IPSW processing. Table 4 shows that adolescents in the “mismatch—parent higher” group had the highest score for depressive symptoms; this was, significantly as to both, the finding for both males and females. This phenomenon was mainly found among adolescents in the “mismatch—parent higher” group whose parental educational expectations were high school and college. In addition, depressive symptoms were significantly higher among female adolescents in the “mismatch—parent lower” group, as compared to the “match” group, but not among the male adolescents. That is, female adolescents are more likely to develop depressive symptoms than are male adolescents when their parental educational expectations are lower than their own educational expectations.

Table 5 shows that the highest levels of depressive symptoms among adolescents in the “mismatch—parent higher” group were significant in the primary schools, but not in the middle schools. Further analysis revealed that this result was mainly seen in parental educational expectations of adolescents for the high school and college groups. Similarly, higher levels of depressive symptoms among adolescents in the “mismatch—parent lower” group were significant only in primary schools, compared to the “match” group.

The first two columns of Table 6 show that the highest levels of depressive symptoms among adolescents in the “mismatch—parent higher” group are significant only in the urban group, and not in the rural group. The last two columns of Table 6 show that, compared to the “match” group, in the “mismatch—parent higher” group, with parental educational expectations of high school, the highest levels of depressive symptoms were found in both rural and urban adolescents, but with parental educational expectations of college, the significantly high levels of adolescent depressive symptoms were only significant among the urban adolescents.

## 4. Discussion

Using data from a recent large national survey in China, we examine the association between parent–child mismatch in educational expectations and depressive symptoms among adolescents. In addition to the association in the overall sample, we also examined whether and how this association is differentiated among adolescents of different genders, educational levels, and regions.

Regression analyses showed that the “mismatch—parent higher” group had the highest levels of depressive symptoms. This finding is consistent with the previous research, which has documented that higher parental educational expectations relative to adolescents are associated with psychological problems in the adolescents [16,27]. Having parents with higher educational expectations (than their own) can lead to academic stress [35] and lower academic achievement [36,37] among adolescents. In severe cases, it can also lower a child’s self-esteem and confidence. These mismatch—parent higher expectations may be negatively associated with mental health [38]. For example, previous research has shown that when parental educational expectations are higher than those of the ad-olescents, there is a decline in emotional well-being among adolescents. [24]. In addition, when parental educational expectations are higher than children’s educational expectations, the situation often leads to disagreements and quarrels between parents and children which will adversely affect adolescents’ mental health [16].

The stratified regression analyses present results for the subsample by gender, education level, and region. First, in terms of gender, “mismatch—parent higher” was significantly associated with depressive symptoms among both males and female adolescents, which is consistent with the findings from previous studies that the stress of high expectations causes no difference between boys and girls as to depressive symptoms [39]. In addition, in the “mismatch—parent lower” group, females had a significantly higher level of depressive symptoms, as compared to male adolescents. This may be due to the inconsistent self-evaluation standards used among male and female adolescents. Males pay more attention to their internal evaluation, while females pay more attention to their external evaluation. The low expectations of parents for female adolescents may cause negative emotions in female adolescents. Second, in terms of educational level, “mismatch—parent higher” was not associated with depressive symptoms in middle school students, but was associated with depressive symptoms in primary school students. These findings suggest that higher parental educational expectations may be particularly detrimental to the emotional health of primary school students. This is broadly consistent with identity control theory, which suggests that primary school students are more dependent on parental educational expectations than are middle school students, and are more likely to experience negative emotions when their own educational expectations do not match those of their parents [24]. In terms of region, we found higher levels of depressive symptoms among urban adolescents in “mismatch—parent higher” group. It may be that rural adolescents’ positive attitudes towards future educational achievement have a moderating effect on depressive symptoms caused by parents’ high educational expectations. It may also be that most of the parents of rural adolescents need to leave home to work and are not around them, leading to the adolescents’ weak perception of their parents’ high educational expectations. It may also be that urban and rural adolescents relax in different ways after school. Urban adolescents experience greater pressure after school, engaging in activities such as participation in a variety of forms of private tutoring, which may exacerbate the depressive symptoms of these urban adolescents [40]. This finding of increased symptoms among urban students is also inconsistent with existing research findings [22]. The results of this prior study showed that when parental educational expectations were higher, depressive symptoms were significantly present in both urban and rural adolescents. The inconsistency with the present study may be due to the inconsistent treatment of educational-expectation-mismatch. In their study, the higher parental educational expectations group included both “match” and “mismatch—parent high”, as compared to the lower parental educational expectations. Therefore, the results are inconsistent between us.

This study has several limitations. First, this study is based on cross-sectional data. Therefore, the underlying causality of the association between parent–child mismatch and depressive symptoms in adolescents cannot be fully established. Second, assessments of depressive symptoms typically include both somatic and affective dimensions. The measure of depressive symptoms used in the present study was a modified version of the CES-D, which focuses primarily on the affective dimension and is therefore limited in capturing the full range of depressive symptoms. Third, we relied on self-reported measures of depressive symptoms, which may be subject to potential recall or reporting bias. Future research using a more objective instrument to capture this variable would be beneficial in order to improve the reliability and validity of the findings.

Despite these limitations, the policy implications of this study are clear and significant. First, the observed association between parent–child mismatch in educational expectations and depressive symptoms underscores the importance of parents’ careful consideration of their children’s educational expectations when forming their own. In China, the central government has recently emphasized the emotional health of adolescents by mandating that all primary and middle school educational institutions introduce mental health education programs to help students better understand depression and the need to seek professional counseling (National Health and Wellness Commission of China, 2020). However, the influence of the parent–child mismatch in educational expectations on depressive symptoms has long been overlooked. Our findings suggest that future reforms in home education should aim to align parents’ and children’s educational expectations in order to improve adolescents’ emotional health. For example, regular family meetings can be held to enhance communication between parents and children. Furthermore, the differential impacts of parent–child mismatch in educational expectations on depressive symptoms across various educational levels and household groups should be carefully considered when designing targeted policies to more effectively address adolescent depressive symptoms.

## 5. Conclusions

This study provides empirical evidence from China that enhances our understanding of the relationship between parent–child mismatch in educational expectations and adolescent depressive symptoms. Using data from a large-scale national survey of Chinese adolescents, our findings suggest that depressive symptoms are most severe among adolescents in the “mismatch—parent higher” group. Furthermore, these symptoms exhibit variation across different educational levels and according to region status. Importantly, these patterns do not show significant gender-based differences. Future health promotion programs targeting adolescents should be specifically tailored to address the needs of different groups, considering the parent–child mismatch in educational expectations, educational levels, and region status.

## Figures and Tables

**Table 1:** Classification of parent–child mismatch in educational expectations.

Parental Educational Expectations	Child Educational Expectations			
	Below Senior High	Senior High	College	Postgraduate
Below senior high	**Group 1**: Match	Group 3a		**Group 3**
			Mismatch-parent higher	
Senior high			Group 3b	
College	**Group 2**: Mismatch-parent lower			Group 3c
Postgraduate				

**Table 2:** Descriptive statistics.

	Total (N = 1844)	Match (N = 1030)	Mismatch—Parent Lower (N = 247)	Mismatch—Parent Higher (N = 567)	*p*-Value
Depressive symptoms (CES-D8)	4.27 (3.51)	4.01 (3.44)	4.20 (3.66)	4.76 (3.53)	<0.001
Parent–child mismatch in educational expectations					<0.001
Match	55.86 (1030)				
Mismatch—parent lower	13.39 (247)				
Mismatch—parent higher	30.75 (567)				
With parental education as senior high	2.39 (44)				
With parental education as college	19.74 (364)				
With parental education as postgraduate	8.62 (159)				
Age (in years)	12.40 (1.68)	12.42 (1.65)	11.97 (1.69)	12.57 (28.04)	<0.001
Gender					<0.001
Male	52.87 (975)	47.48 (489)	60.73 (150)	59.26 (336)	
Female	47.13 (869)	52.52 (541)	39.27 (97)	40.74 (231)	
Parental education in years	9.49 (3.62)	9.62 (3.61)	9.62 (3.67)	9.20 (3.60)	0.076
Per capital family income: logged	9.50 (0.99)	9.54 (0.97)	9.49 (0.93)	9.44 (1.05)	0.160
Grade	3.5 (1.70)	3.53 (1.73)	3.7 (1.54)	3.35 (1.70)	0.014
Academic achievement: Math	2.68 (1.04)	2.77 (1.05)	2.65 (0.99)	2.54 (1.04)	<0.001
Academic achievement: Chinese	2.69 (0.94)	2.79 (0.92)	2.64 (0.91)	2.52 (0.96)	<0.001
Region					0.712
Rural	57.38 (1058)	56.60 (583)	57.49 (142)	58.73 (333)	
Urban	42.62 (786)	43.40 (447)	42.51 (105)	41.27 (234)	

Note: Data are presented as mean (SD) for continuous measures, and n (%) for categorical measures.

**Table 3:** Regression of depressive symptoms on parent–child mismatch in educational expectations.

	Model 1		Model 2	
	Coefficient	SE	Coefficient	SE
Parent–child mismatch in expectations				
Match (reference)				
Mismatch—parent lower	0.18	(0.27)	0.18	(0.27)
Mismatch—parent higher	0.59 **	(0.19)		
With parental education as senior high			2.25 **	(0.79)
With parental education as college			0.65 **	(0.23)
With parental education as postgraduate			0.26	(0.35)
R^2^	0.005		0.025	
F	4.75 **		3.74 **	
N	1844		1831	

Note: Estimations were adjusted by the inverse propensity-score weighting. The propensity score of being assigned into a certain category of parent–child mismatch was predicted by a set of covariates, including age, gender, parental education, family income, grade, and academic performance in math and Chinese. ** *p* < 0.01.

**Table 4:** Regression estimates of parent–child mismatch in educational expectations: by gender.

	Model 1		Model 2	
	Male	Female	Male	Female
Parent–child mismatch in educational expectations				
Match (reference)				
Mismatch—parent lower	−0.39	0.80 +	−0.39	0.80 +
	(0.30)	(0.45)	(0.30)	(0.45)
Mismatch—parent higher	0.45 +	0.74 *		
	(0.24)	(0.30)		
With parental education as senior high			1.77 *	2.85 *
			(0.89)	(1.29)
With parental education as college			0.53 +	0.78 *
			(0.29)	(0.37)
With parental education as postgraduate			−0.07	0.63
			(0.40)	(0.58)
R^2^	0.011	0.009	0.029	0.031
F	3.5 *	3.94 *	2.68 *	2.78 *
N	975	869	968	863

Note: Estimations were adjusted by the inverse propensity-score weighting. The propensity score of being assigned into a certain category of parent–child mismatch was predicted by a set of covariates, including age, gender, parental education, family income, grade, and academic performance in math and Chinese. Standard errors in parentheses; + *p* < 0.1, * *p* < 0.05.

**Table 5:** Regression estimates of parent–child mismatch in educational expectations: by educational level.

	Model 1		Model 2	
	Primary School	Middle School	Primary School	Middle School
Parent–child mismatch in educational expectations				
Match (reference)				
Mismatch—parent lower	0.61 +	−0.43	0.61 +	−0.43
	(0.36)	(0.42)	(0.36)	(0.42)
Mismatch—parent higher	0.87 ***	0.20		
	(0.25)	(0.28)		
Parental education as senior high			2.74 **	1.12
			(0.88)	(1.28)
Parental education as college			0.88 **	0.33
			(0.31)	(0.34)
Parental education as postgraduate			0.61	−0.22
			(0.43)	(0.59)
R^2^	0.010	0.006	0.040	0.011
F	6.49 **	1.09	4.69 ***	0.91 *
N	1075	769	1066	765

Note: Estimations were adjusted by the inverse propensity-score weighting. The propensity score of being assigned into a certain category of parent–child mismatch was predicted by a set of covariates, including age, gender, parental education, family income, grade, and academic performance in math and Chinese. Standard errors in parentheses; + *p* < 0.1, * *p* < 0.05, ** *p* < 0.01, *** *p* < 0.001.

**Table 6:** Regression estimates of parent–child mismatch in educational expectations: by region.

	Model 1		Model 2	
	Rural	Urban	Rural	Urban
Parent–child mismatch in educational expectations				
Match (reference)				
Mismatch—parent lower	0.37	−0.08	0.37	−0.08
	(0.37)	(0.39)	(0.37)	(0.39)
Mismatch—parent higher	0.35	0.91 **		
	(0.24)	(0.31)		
Parental education as senior high			1.40 *	3.54 *
			(0.68)	(1.56)
Parental education as college			0.37	1.03 **
			(0.29)	(0.38)
Parental education as postgraduate			−0.09	0.70
			(0.39)	(0.62)
R^2^	0.002	0.016	0.013	0.056
F	1.27	4.79 *	1.57	3.38 **
N	1058	786	1049	782

Note: Estimations were adjusted by the inverse propensity-score weighting. The propensity score of being assigned into a certain category of parent–child mismatch was predicted by a set of covariates, including age, gender, parental education, family income, grade, and academic performance in math and Chinese. Standard errors in parentheses; * *p* < 0.05, ** *p* < 0.01.

## Data Availability

The dataset supporting the conclusions of this article is available on the website of China Family Panel Studies (CFPS). (Hyperlink to dataset at http://www.isss.pku.edu.cn/cfps/ (accessed on 1 September 2023).
